# Burnout and workload among hematologists in the Community of Madrid (Spain): a survey based on the Copenhagen burnout inventory

**DOI:** 10.3389/fpubh.2026.1905977

**Published:** 2026-08-24

**Authors:** A. Alegre-Amor, R. Bailén-Almorox, M. Bastos-Oreiro, M.-A. Foncillas, P. Martínez-Barranco, M. Morado-Arias, L. Pardo-Gambarte, C. Serí-Merino, L. Solán-Blanco, I. Barber-Turro, A. Cedillo, L. Benito-Parra, V. Jiménez-Yuste, J.-A. Hernández-Rivas, on behalf of Asociación Madrileña de Hematología y Hemoterapia (AMHH)

**Affiliations:** 1 Hospital Universitario de La Princesa, Madrid, Spain; 2 Hospital General Universitario Gregorio Marañón, Madrid, Spain; 3 Hospital Universitario Infanta Leonor, Madrid, Spain; 4 Hospital Universitario Fundación Alcorcón, Madrid, Spain; 5 Hospital Universitario La Paz, Madrid, Spain; 6 Hospital Universitario Fundación Jiménez Díaz, Madrid, Spain; 7 Hospital Central de la Defensa Gómez Ulla, Madrid, Spain; 8 LetsFocusNow, Madrid, Spain; 9 Asociación Madrileña de Hematología y Hemoterapia, Madrid, Spain; 10 Hospital Universitario de Getafe, Madrid, Spain; 11 Universidad Complutense, Madrid, Spain

**Keywords:** burnout, CBI survey, hematology, occupational health, workload

## Abstract

**Background:**

Burnout is highly prevalent among hematologists, with rates reported ranging from 40 to 50%, driven by the unique combination of high clinical complexity, frequent exposure to life-threatening conditions, and the rapid evolution of therapeutic strategies. This study aimed to assess the prevalence and associated factors of burnout among hematologists working in the Community of Madrid, and to examine its impact on their professional performance and personal and social well-being.

**Methods:**

Cross-sectional observational study based on an online self-administered survey. Hematologists across all professional settings were invited to participate by completing the Copenhagen Burnout Inventory (CBI). Overall CBI scores and subscale scores (personal, work-related, and patient-related burnout) were calculated. Subgroup analyses were performed according to demographic and professional variables.

**Results:**

Forty percent (129/320) of the invited hematologists participated in the survey. Respondents reported significant symptoms of exhaustion and an increased risk of burnout, particularly in the work-related domain. Sixty percent of the respondents often or always experienced fatigue, and 23% frequently felt they “cannot take it anymore.” Eighty-four percent reported that work was emotionally exhausting at least sometimes, and 39% frequently ended the workday exhausted. In addition, 55% reported having little energy for family and friends. Patient-related burnout was generally moderate, yet over half strongly agreed that working with patients is hard. Physical or verbal aggression increased from 23 to 30% between 2023 and 2024 (*n* = 44). Mean CBI scores were 43.93 (SD 18.6) for personal, 46.29 (SD 16.0) for work-related, and 45.25 (SD 21.2) for patient-related burnout, with a total score of 45.16 (SD 15.2); 28% of participants reached at least a moderate level of exhaustion on the total score. Thirty-five percent of respondents (18 of the 53 who answered this item) required psychological or psychiatric consultation in 2024.

**Conclusion:**

Hematologists in the Community of Madrid reported relevant symptoms of exhaustion across all dimensions assessed. Workload was the factor most frequently cited by respondents as contributing to burnout. These findings underscore the need for organizational interventions to reduce workload and protect work-life balance.

## 1 Background

Burnout is a syndrome of occupational exhaustion that arises when the adaptive response to a demanding work environment fails. According to the WHO ICD-11 classification, burnout is an occupational phenomenon that influences health status but is not classified as a disease or medical condition. This distinction has been highlighted to emphasize the need to establish clear boundaries to avoid misusing the term burnout to describe any discomfort at work (1). In particular, burnout should not be confused with depression, with the “compassion fatigue” of caregivers (2, 3), or with simple work fatigue. Burnout requires a stressful environment, a failure of defense mechanisms, and the appearance of a constellation of symptoms centered on emotional exhaustion, decreased sense of professional efficacy, reduced autonomy, and disconnection from the professional community (4).

Physicians are at high risk of burnout (4, 5) due to multifactorial causes, including increased workload (more patients with fewer staff), financial constraints (for staff, facilities, and treatments), and the continuous need for change to meet new demands from healthcare systems and patients (6). There are numerous published reports of burnout among physicians, as well as reports on each specific medical specialty. Hematology is a uniquely multifaceted specialty encompassing malignant and non-malignant disease, pediatric and adult populations, laboratory and hemostasis tasks, emergency care, and high-complexity therapeutics. Hematologists are exposed to significant emotional and psychological stressors at work (7) and therefore at risk of burnout (8–11).

The *Asociación Madrileña de Hematología y Hemoterapia* (AMHH) is a professional society whose members are hematologists (predominantly treating adult but also pediatric patients) working across either public hospitals under a single regional health authority or private centers, all within the Community of Madrid (12). The AMHH encompasses all facets of hematology practice, including malignant and non-malignant hematology, laboratory, hemostasis, bone marrow transplantation, CAR-T, transfusion medicine, and apheresis units. The AMHH conducted this study to analyze workload and the prevalence of burnout among hematologists in the 32 hospitals in the Community of Madrid. With approximately 7,000,000 inhabitants and a hematologist-to-population ratio of 4.0 per 100,000 in 2025 (down from 4.3 in 2021, the Spanish national average), this region serves as a meaningful benchmark and pilot model for assessing hematological workload. Participating hematologists work across departments of varying complexity (primary, secondary, and tertiary care settings), supporting the broad applicability of our findings and their potential benefits to clinicians, laboratories, and patients.

With this background in mind, we designed a survey-based study to assess the prevalence and associated factors of burnout among hematologists working in the Community of Madrid, and to examine its impact on their professional performance and personal and social well-being.

## 2 Methods

### 2.1 Hypothesis and objectives

We postulated that a progressive increase in workload over time was exposing hematologists working in the Community of Madrid to a significant risk of burnout. The objective of this study was to quantify this risk and identify the workload factors most strongly associated with burnout.

### 2.2 Participants

All the hematologists working in the Community of Madrid who are members of the AMHH were invited to participate in the survey. No exclusion criteria were applied, and no participant was excluded: the survey was distributed to the whole target population, and every returned questionnaire was analyzed.

### 2.3 Study design

This was a cross-sectional observational study based on an online self-administered survey. The questionnaire was distributed to the 320 hematologists and hematology residents working in the Community of Madrid through the AMHH, which initiated and coordinated the study. Participation was voluntary. A self-administered anonymous questionnaire was provided to potential participants. The project began with the coordinators designing a 39-question questionnaire, divided into four thematic blocks: workload, distribution of working time, perception of the work environment, and level of professional burnout. The questionnaire included the Copenhagen Burnout Inventory (CBI) (13), as a standardized, publicly available tool internationally recognized for its psychometric validity, designed to measure burnout in three areas: personal, work-related, and patient-related, with demonstrated psychometric robustness in the Spanish population, along with additional questions on professional profile, workload, and work-related variables. A previous cross-sectional study was conducted with Spanish workers in education, social work, healthcare, and industry to assess the instrument’s reliability, validity, and usefulness in the Spanish context (14). The results demonstrate that the Spanish CBI has high internal consistency and high psychometric robustness in the Spanish population (14). The burnout assessment consisted of the 19 original CBI items, taken from the Spanish version of the instrument14, organized into three domains: personal burnout (6 items), work-related burnout (7 items), and patient-related burnout (6 items). No burnout item was developed by the authors; the remaining sections of the questionnaire (professional profile, workload, working conditions, and organizational variables) were developed by the authors for this study and are independent of the CBI. The original CBI items were retained, but the response format differed from that of the Spanish validation study, which used a five-category scale for the three domains. In our questionnaire, the personal and work-related domains were administered with a four-category frequency scale (Always = 100, Frequently = 66.67, Sometimes = 33.33, Never/Almost never = 0) and the patient-related domain with a five-category degree scale (very high degree = 100, high degree = 75, a little = 50, low degree = 25, very low degree = 0). We do not claim that this response format has been separately validated. Both formats were converted to the standard 0 to 100 CBI metric: responses were averaged within each subscale and rounded to the nearest whole number. Although all scores are expressed on the standard 0 to 100 metric, the difference in response format should be taken into account when comparing our results with studies using the original CBI response scale. The three domain scores, each given the same weight (one-third) in the overall weighting, provided the basis for calculating an overall score (total burnout score) on a scale of 0 to 100. Based on the CBI scoring guidelines13, the following bands were used as interpretive categories to describe the severity of exhaustion, and not as diagnostic cut-off values: 0–49: low level of exhaustion; 50–74: moderate exhaustion; 75–89: high exhaustion; 90–100: very high exhaustion.

### 2.4 Statistical analysis

Descriptive analyses were performed. Continuous variables were described as means and range, and categorical variables as frequencies and percentages. To assess associations between demographic and occupational variables and CBI scores, normality was first assessed using the Shapiro–Wilk test. For variables with two groups (center complexity and primary care activity), group differences were compared using a Student’s t-test if both groups were normally distributed, or the Mann–Whitney U test otherwise. For variables with more than two groups (professional position, years of experience, age group, and work setting), differences were compared using one-way ANOVA if normality was confirmed in all groups, or the Kruskal-Wallis test otherwise. Statistical significance was set at *p* < 0.05. The CBI block was completed by 82 of the 129 respondents. No imputation was applied: subscale scores and item percentages were computed by available-case analysis over valid responses, so that the number of participants contributing to each score was 82 for personal and work-related burnout and 79 for patient-related and total burnout. Non-response to the burnout block was assessed by comparing completers and non-completers across the available demographic and occupational variables, using the chi-square test or Fisher’s exact test as appropriate. Means are reported with standard deviations and 95% confidence intervals, proportions with 95% confidence intervals (Wilson method), and statistically significant group differences with the corresponding mean difference, its 95% confidence interval, and Cohen’s d as a measure of effect size. Comparisons involving small subgroups are reported as exploratory.

### 2.5 Ethics statement

This study constitutes research involving human participants but did not undergo formal review by an institutional Research Ethics Committee. It was a fully anonymous, voluntary, self-administered survey of professional opinion among physicians about their own working conditions, with no patients, clinical data, biological samples, or identifiable personal data involved. The investigators determined that, under the applicable Spanish regulations on biomedical research (Ley 14/2007) and data protection (Regulation (EU) 2016/679 and Ley Orgánica 3/2018), a survey of this nature is not subject to mandatory review by a Research Ethics Committee; this was not confirmed by a committee or other competent authority. Formal approval and written informed consent were therefore not obtained; submission of the anonymous questionnaire was taken as implied consent to participate.

## 3 Results

### 3.1 Professional and occupational characteristics of the participants

The CBI questionnaire was distributed between March and April 2025 to 320 hematologists and hematology residents, of whom 129 (40%) responded. Most hematologists (107 out of 129; 83%) worked at centers funded by public insurance. Two-thirds of hematologists (83 out of 129; 65%) worked in tertiary care centers that perform high-complexity therapeutics (bone marrow transplantation and CAR-T), while only 3 (2%) worked in low-complexity centers. Sixteen hematologists (12%) held senior clinical management positions (Head of Department or Head of Section). Twelve of them (9%) were hematology residents. The largest age group (59; 46%) was 35–55 years old. The most frequent range of professional experience was 5 to 15 years (49; 38%). The most frequent work settings were clinical, alone (55; 43%) or clinical combined with laboratory and/or transfusion medicine (56; 43%). Only 9% worked exclusively in the laboratory and 5% exclusively in transfusion medicine. Supplementary Table S1 and Supplementary Figure S1 present additional data on the participants’ professional and occupational characteristics.

### 3.2 Workload

Patient care was the primary work activity for 94% of the 101 participants who answered this item. The most common reported weekly patient load was 26–50 (33%). Notably, four participants (4%) managed more than 100 patients per week. Teaching duties and management each accounted for 10% of the hematologists’ working time. Research accounted for 5% of the workload. Ninety-seven (76%) of the 127 hematologists who answered this item worked extra hours beyond their regular work hours, with 54% spending more than 10 unpaid extra hours per week. These overtime hours were mainly dedicated to patient care (46% of the participants), research (31%), and management (12%). Forty-five respondents (44%) had access to, and engaged in, telework. Seventy-five hematologists (58%) perform 24-h on-call shifts and may be on call at the hospital, on call outside the hospital, or in both on some days, on a monthly schedule. The most common frequency was one to five shifts per month: at the hospital, reported by 35 hematologists (27%), outside the hospital (12%), and combining both modalities (10%). Supplementary Table S2 and Supplementary Figure S2 present additional data on participants’ workload. Supplementary Figures S3–S6 show data on time distribution by work area and the future perspective of variation in the time distribution by work area in both clinical and non-clinical settings.

### 3.3 Burnout

#### 3.3.1 Personal burnout

Symptoms of personal burnout were characterized by physical and psychological exhaustion, thoughts of incapacity (‘I cannot take it anymore’), and an overall sense of weakness. The data revealed a high prevalence of these symptoms: 47% of respondents reported experiencing some of them “sometimes,” and 37% reported experiencing them “frequently.” Only 12% of the participants reported never experiencing these signs of personal exhaustion. At the higher end of the severity scale, the most common symptoms were “fatigue” and “psychological exhaustion,” which occurred “frequently” or “always” in 66 and 56% participants, respectively. The thought “I cannot take it anymore” occurred “frequently” or “always” in 23% of the respondents. The personal burnout domain was answered by 82 participants. Table 1 and Supplementary Figures S7, S8 show the full data, with the valid n for each item, on how frequently respondents experience symptoms associated with personal burnout.

**Table 1:** Frequency of symptoms of personal burnout.

*N* = 129	Never *n* (%)	Sometimes *n* (%)	Frequently *n* (%)	Always *n* (%)	NA *n* (%)
How frequently are you tired? (*n* = 82)	1 (1)	27 (33)	49 (60)	5 (6)	*47 (36)*
How frequently are you physically exhausted? (*n* = 82)	7 (9)	40 (49)	31 (38)	4 (5)	*47 (36)*
How frequently are you psychologically exhausted? (*n* = 82)	4 (5)	32 (39)	41 (50)	5 (6)	*47 (36)*
How frequently do you think “I cannot take it anymore”? (*n* = 81)	13 (16)	49 (60)	18 (22)	1 (1)	*48 (37)*
How frequently are you exhausted? (*n* = 82)	7 (9)	41 (50)	31 (38)	3 (4)	*47 (36)*
How frequently do you feel weak and prone to illness? (*n* = 82)	28 (34)	41 (50)	12 (15)	1 (1)	*47 (36)*
Total personal burnout % Mean *n* = 6	12	47	37	4	—

Percentages for response options are calculated over the number of respondents per item; the valid n is given for each item. NA: not answered; percentage calculated over total sample (*n* = 129).

Mean values in the “Total” row are calculated as the average of response percentages across the n items of the corresponding block.

#### 3.3.2 Work burnout

The most common work-related burnout symptom was feeling that work was emotionally exhausting, reported by all respondents at least sometimes (40% sometimes, 44% frequently, 16% always). No respondent reported never experiencing this feeling. Nine percent experienced burnout constantly (“always”). Twenty-nine percent of the respondents reported feeling frequently burned out at work, and 39% reported frequently ending the workday exhausted. Two-thirds of hematologists (67%) “sometimes” or “frequently” felt that “every hour of work is exhausting.” Fifty-four percent of respondents reported sometimes or frequently having little energy left for family and friends outside of work. The work-related burnout domain was answered by 82 participants. Table 2 and Supplementary Figures S7, S9 show the full data, with the valid n for each item, on how frequently respondents experience the symptoms and feelings associated with work burnout.

**Table 2:** Frequency of symptoms of work-related burnout.

*N* = 129	Never *n* (%)	Sometimes *n* (%)	Frequently *n* (%)	Always *n* (%)	NA *n* (%)
Is your work emotionally exhausting? (*n* = 82)	0 (0)	33 (40)	36 (44)	13 (16)	*47 (36)*
Do you feel burned out from your job? (*n* = 82)	6 (7)	45 (55)	24 (29)	7 (9)	*47 (36)*
Do you feel frustrated with your job? (*n* = 82)	13 (16)	42 (51)	23 (28)	4 (5)	*47 (36)*
Do you feel exhausted at the end of the workday? (*n* = 82)	3 (4)	35 (43)	32 (39)	12 (15)	*47 (36)*
Do you feel exhausted in the morning with the thought of another day at work? (*n* = 82)	16 (20)	45 (55)	15 (18)	6 (7)	*47 (36)*
Do you feel like every hour of work is exhausting? (*n* = 82)	27 (33)	42 (51)	13 (16)	0 (0)	*47 (36)*
Do you have enough energy for your family and friends in your free time? (*n* = 82)	3 (4)	25 (30)	45 (55)	9 (11)	*47 (36)*
Total work-related burnout % Mean *n* = 7	12	47	33	9	—

Percentages for response options are calculated over the number of respondents per item; the valid n is given for each item. NA: not answered; percentage calculated over total sample (*n* = 129).

Mean values in the “Total” row are calculated as the average of response percentages across the *n* items of the corresponding block.

#### 3.3.3 Burnout linked to direct care of patients

Overall, most respondents reported moderate levels of patient-related exhaustion: 30% felt it “a little,” and 22% felt it “in a high degree.” More than half of the participants (51%) strongly agreed with the statement that “working with patients is hard.” Frustration and uncertainty about how long participants could continue working with patients appeared less pronounced, although 18 and 21%, respectively, reported coping with these feelings at high or very high degrees. Respondents were asked to retrospectively report incidents in both 2023 and 2024. Of note, among the 44 hematologists who answered this item, 30% reported experiencing physical or verbal aggression in 2024, compared with 23% in 2023. The rates of formal complaints followed a similar pattern, rising from 24% in 2023 (*n* = 49) to 36% of the respondents in 2024 (*n* = 53). The patient-related burnout domain was answered by 79 participants; the three respondents who did not answer it were laboratory- or mixed-profile hematologists without direct patient contact. Table 3 and Supplementary Figures S7, S10 summarize, with the valid n for each item, hematologists’ perceptions of these feelings associated with direct patient care.

**Table 3:** Symptoms of patient-related burnout.

*N* = 129	In a very low degree *n* (%)	In a low degree *n* (%)	A little *n* (%)	In high degree *n* (%)	In a very high degree *n* (%)	NA *n* (%)
Is it hard to work with patients? (*n* = 79)	3 (4)	8 (10)	28 (35)	32 (41)	8 (10)	*50 (39)*
Is it frustrating to work with patients? (*n* = 78)	13 (17)	28 (36)	23 (29)	13 (17)	1 (1)	*51 (40)*
Does working with patients drain your energy? (*n* = 79)	4 (5)	9 (11)	29 (37)	28 (35)	9 (11)	*50 (39)*
Do you feel like you give more than you get when working with patients? (*n* = 78)	9 (12)	19 (24)	25 (32)	16 (21)	9 (12)	*51 (40)*
Are you tired of working with patients? (*n* = 79)	23 (29)	32 (41)	19 (24)	4 (5)	1 (1)	*50 (39)*
Do you ever wonder how much longer you will be able to keep working with patients? (*n* = 79)	21 (27)	21 (27)	20 (25)	12 (15)	5 (6)	*50 (39)*
Total patient-related burnout % Mean *n* = 6	15	25	31	22	7	—

Degree of agreement.

Percentages for response options are calculated over the number of respondents per item; the valid *n* is given for each item. NA: not answered; percentage calculated over total sample (*n* = 129).

Mean values in the “Total” row are calculated as the average of response percentages across the n items of the corresponding block.

#### 3.3.4 Total burnout score and need for mental health consultation

Based on the questionnaire responses, the average scores on the CBI scale were 43.93 for personal burnout (SD 18.6; 95% CI 39.8–48.0; *n* = 82), 46.29 for work-related burnout (SD 16.0; 95% CI 42.8–49.8; *n* = 82), and 45.25 for patient-related burnout (SD 21.2; 95% CI 40.5–50.0; *n* = 79). These three scores yielded a mean total CBI score of 45.16 (SD 15.2; 95% CI 41.8–48.6; *n* = 79). Applying the pre-defined interpretive bands, 28% of participants (22/79; 95% CI 19.2–38.6) reached at least a moderate level of exhaustion on the total score, and 3% (2/79) reached a high level. The corresponding proportions by domain were 48% (39/82) for personal, 32% (26/82) for work-related, and 43% (34/79) for patient-related burnout.

Thirty-five percent of the hematologists working in the Community of Madrid needed consultation with a psychologist or psychiatrist in 2024, the year immediately preceding the survey (18 of the 53 who answered this item), compared with 25% in 2023 (12 of 52).

#### 3.3.5 Perceived influence of work-related variables on burnout

We also analyzed the impact of various work variables on hematologists’ burnout levels. Workload was the factor most frequently cited by respondents as contributing to burnout, with 112 (88%) rating its impact “to a high degree” (49%) or “very high degree” (39%). Only 2% reported that workload had little influence on their burnout levels. Other work variables with relevant percentages of combined “high degree” or “very high degree” impact on burnout were salary (53%), interpersonal relationships with the department team (46%), and department facilities and resources (41%). Table 4 shows the impact of various work variables on burnout levels, as perceived by the responding hematologists.

**Table 4:** Work variables impacting burnout level.

*N* = 129	In a very low degree *n* (%)	In a low degree *n* (%)	A little *n* (%)	In high degree *n* (%)	In a very high degree *n* (%)	NA *n* (%)
Salary (*n* = 81)	4 (5)	7 (9)	27 (33)	35 (43)	8 (10)	*48 (37)*
Relationship with the department staff (*n* = 82)	7 (9)	13 (16)	24 (29)	27 (33)	11 (13)	*47 (36)*
Facilities and resources of the department (*n* = 82)	8 (10)	13 (16)	27 (33)	28 (34)	6 (7)	*47 (36)*
Workload (*n* = 82)	0 (0)	2 (2)	8 (10)	40 (49)	32 (39)	*47 (36)*
Relationship with superiors (*n* = 82)	9 (11)	16 (20)	26 (32)	22 (27)	9 (11)	*47 (36)*
Relationship with other hospital departments (*n* = 82)	9 (11)	26 (32)	28 (34)	15 (18)	4 (5)	*47 (36)*
Recognition within my department (*n* = 82)	7 (9)	23 (28)	23 (28)	22 (27)	7 (9)	*47 (36)*
Recognition within my hospital (*n* = 81)	15 (19)	23 (28)	23 (28)	14 (17)	6 (7)	*48 (37)*
Recognition in the scientific setting outside my hospital (*n* = 82)	20 (24)	25 (30)	23 (28)	9 (11)	5 (6)	*47 (36)*
Social recognition (*n* = 82)	17 (21)	22 (27)	26 (32)	14 (17)	3 (4)	*47 (36)*
Recognition of Hematology among all specialties (*n* = 79)	16 (20)	21 (27)	27 (34)	15 (19)	0 (0)	*50 (39)*

Degree of agreement.

Percentages for response options are calculated over the number of respondents per item; the valid n is given for each item. NA: not answered; percentage calculated over total sample (*n* = 129).

#### 3.3.6 Association between demographic characteristics and burnout features

Working in high-complexity tertiary care hospitals was the only variable significantly associated with higher burnout scores, specifically for personal burnout (47.8 vs. 38.4; mean difference 9.4, 95% CI 1.2–17.6; Cohen’s d = 0.52; Student’s t-test *p* = 0.025) and total burnout (47.9 vs. 40.0; mean difference 7.8, 95% CI 1.0–14.7; Cohen’s d = 0.53; Mann–Whitney U test *p* = 0.01), both representing a moderate effect size. In contrast, professional position, years of experience, age group, work setting, and primary work activity did not show a statistically significant association with burnout levels. Participants who completed the burnout block (*n* = 82) and those who did not (*n* = 47) did not differ in center funding, center complexity, age, or work setting; in particular, they did not differ in center complexity, the only variable associated with burnout in our data. Residents were more likely than staff specialists to leave the burnout block incomplete (Fisher’s exact test, *p* = 0.030).

## 4 Discussion

The results of this study show that hematologists working in the Community of Madrid exhibit relevant levels of burnout, with mean CBI scores in the upper part of the low band and approaching the moderate band of the pre-defined interpretive categories (personal: 43.93, work-related: 46.29, patient-related: 45.25). Workload was the factor most frequently cited by respondents as contributing to burnout (88% reported high or very high impact). A specialty-specific factor is the multifaceted nature of their work, which further increases the risk. The significant workload faced by hematologists extends beyond direct clinical care to include laboratory work, transfusion medicine, teaching, research, and administrative tasks. Feelings of exhaustion were frequently reported by hematologists, a core self-reported dimension of burnout. When asked about perceived stressors, respondents identified workload as the primary contributor, followed by low salary as a relevant concern. This multifaceted role generates multiple demands from coworkers, patients, and families, extending beyond conventional patient care and increasing the risk of burnout (10). While workload is a clearly modifiable factor, the stress associated with caring for seriously ill patients and the multifaceted nature of hematology work require organizational-level interventions such as adequate staffing, integration of advanced practice providers, structured psychological support programs, and workflow optimization to mitigate their impact on burnout. The administrative workload (drug or reagent expenditures justification, supply orders, reports for hospital management, ambulance requests) deserves special mention, as it represents an excessive burden that is not commensurate with physicians’ professional status, further reducing the time available for direct patient care, and is perceived as particularly burdensome by hematologists (15). The study sample is appropriate, comprising mainly hematology and hemotherapy specialists, most of whom work primarily in the public sector and are largely based at highly complex centers. Of note, the respondents are balanced between those dedicated exclusively to clinical tasks and those with mixed roles, reflecting the diversity of the hematology workforce worldwide.

Our findings should be compared with those of previous studies. A prior report (14) compared burnout risk across 12 professional healthcare categories (general practice physicians, resident physicians, nurses, social workers, administrative staff, among others), considering customer (patient)-linked and work-related burnout. When these results are compared with our participants, higher scores for both dimensions were observed in our series, surpassing 11 of those categories, with only the administrative group surpassing them. In turn, the validation study of the CBI questionnaire examined various populations with average total burnout scores of 32.00 for the general public and 36.50 for physicians. A separate study that assessed psychiatrists and internal medicine specialists found a higher mean score of 41.50. Our average total burnout score of 45.16 points in hematologists is above these figures, consistent with the relevant risk of burnout among our participants. Comparing our findings with studies performed specifically with hematologists, the burnout prevalence observed in our sample (CBI total score: 45.16) is consistent with, though slightly higher than, rates reported in recent international studies. Gürcan et al. (7) found that 35% of Turkish hematologists experienced high emotional exhaustion, while Lee et al. (8) reported a 29% burnout rate among US hematologists-oncologists. The Italian study by Bressi et al. (16) found 32% of hemato-oncology professionals with high emotional exhaustion. These comparisons suggest that burnout among hematologists in the Community of Madrid is at least as prevalent as in other international settings, and potentially higher. Furthermore, our results are consistent with those of an international survey conducted by the European Hematology Association (EHA), which reported similarly relevant levels of burnout across various countries. Of note, Spain ranks second, after Italy, in the number of hematologists who responded to the survey. In particular, our findings are consistent with the European survey (17), in which workload was likewise the factor most frequently reported as contributing to burnout among hematologists. In addition, the broader significance of these findings lies in the negative impact of burnout on the quality of patient care, as highlighted by a meta-analysis specifically designed to address this issue (18). These comparisons should be interpreted with caution because of methodological differences among the studies: the instruments used differ (the CBI in the validation study and in our survey, and the Maslach Burnout Inventory in the studies by Gürcan et al. (7), Lee et al. (8), and Bressi et al. (16), which report the proportion of participants with high emotional exhaustion rather than a mean 0 to 100 score), the populations differ in professional composition and case mix, and the healthcare-system contexts differ in staffing, workload, and organizational structure. In addition, the reference values drawn from the Spanish validation study were obtained with a five-category response format, whereas our personal and work-related domains used a four-category format; accordingly, these comparisons are presented as approximate rather than direct.

In the context of physician burnout, it is noteworthy that organizational factors, particularly workload, compensation, and interpersonal relationships within the department, were identified as the most significant contributors, rather than direct patient care tasks per se. This finding aligns with evidence suggesting that approximately 80% of burnout is attributable to organizational rather than individual factors. It is also notable that most respondents report having little energy for family and friends outside work, suggesting a profound impact of burnout on work-life balance and personal and social well-being. This is frequently accompanied by detachment from work, a sense of ineffectiveness, and feelings of exhaustion or cynicism (19). In addition, hematologists spend a significant amount of time on work activities outside their official working hours.

We selected the CBI among other available instruments, and this choice warrants justification, given that the Maslach Burnout Inventory (MBI) (20) remains the most widely used instrument in the literature. As previously noted (21), most studies have relied on the MBI, which defines burnout as emotional exhaustion, depersonalization, and reduced personal accomplishment. However, a systematic review of 182 studies from 45 countries (22) identified substantial heterogeneity, partly due to the lack of consensus on diagnostic criteria and to methodological limitations of the MBI, including its inability to distinguish between patient−/client-related and non-patient−/client-related burnout. In line with that review’s recommendations, we chose the CBI supported by evidence on Portuguese physicians (23) and by the validation of the Spanish version of this questionnaire (14). Furthermore, this public-domain questionnaire has demonstrated robust psychometric properties in the Spanish population (14). Together, this evidence supports the use of the CBI as a methodologically sound instrument for assessing burnout among AMHH members.

### 4.1 Limitations

The survey was completed by 40% of those to whom it was offered, raising the possibility of selection bias, as those who chose to participate may have been more sensitized to burnout-related issues. In addition, non-respondents may have been too overwhelmed with workload to engage with the survey, potentially underestimating the true prevalence of burnout. The study sample is drawn from a clearly defined urban area, the city of Madrid, and its immediate surroundings. This geographic specificity limits extrapolation to other regions, particularly rural or mixed rural–urban areas, where different lifestyles and healthcare systems may predominate and a greater degree of professional isolation among hematologists, together with more limited access to continuing education, may have influenced the outcomes. A further limitation concerns the completeness of the burnout data. The CBI block was completed by 82 of the 129 respondents, so that the burnout estimates rest on 82 participants (79 for the patient-related and total scores) rather than on the full sample. Completers and non-completers did not differ in center complexity, the only variable associated with burnout in our data, which makes a substantial bias in the reported scores unlikely; however, residents were more likely to leave the burnout block incomplete, and the CBI estimates therefore best represent staff specialists. The retrospective items on aggression, formal complaints, and mental-health consultation were answered by fewer participants (*n* = 44 to 53), and these figures are correspondingly less precise. Because these items refer to events in 2023 and 2024 and were reported retrospectively, they are also subject to recall bias. Gender was not recorded in this survey. The questionnaire was designed to be strictly anonymous and, in a small and well-defined professional community such as ours, recording gender together with hospital, position, age, and years of experience could have allowed indirect re-identification of individual respondents. As a consequence, an analysis of burnout scores by gender, a relevant variable in burnout research, is not possible with the present data, and this should be addressed in future studies under a design that preserves anonymity.

### 4.2 Implications and future research

Our findings have implications for healthcare policy. Hospitals’ leadership should monitor and limit excessive working hours, redistribute additional shifts, reinforce teams by ensuring safe staffing ratios (24, 25), and use organizational tools to streamline workflows. These decisions require strategic workforce planning, protected time for non-clinical tasks, contract stability, investment in roles that reduce clinicians’ administrative burden, and recognition of burnout as a major occupational risk (26). Patient safety will benefit from all these measures (27–31). Our findings highlight the need for comprehensive burnout-prevention strategies, consistent with previous literature (26, 30–32). Emotional management and self-care, along with training in stress management, resilience, and early burnout detection, serve both preventive and therapeutic purposes (30). The burden of burnout among hematologists is increasingly recognized, though evidence remains limited compared with other specialties. Future research should evaluate the effectiveness of corrective measures at both the individual and organizational levels (8), as evidence suggests that combining both approaches is more effective than either alone (7). What remains to be established is which strategies prove effective in practice. Longitudinal studies assessing the real-world impact of structural interventions would represent a meaningful step forward.

## 5 Conclusion

Hematologists working in the Community of Madrid report relevant symptoms of exhaustion across all assessed dimensions, with work-related burnout slightly exceeding patient-related burnout, and scores higher than the published reference values for the general population and physicians in other specialties. Workload was the factor most frequently cited by respondents as contributing to burnout, possibly compounded by the multifaceted nature of hematology practice. Burnout extends beyond the workplace, with a profound impact on personal and social well-being, as reflected by the increasing need for mental health support among respondents. Although these findings are limited to an urban setting, they may inform burnout prevention strategies in similar contexts and underscore the need for organizational interventions to reduce workload and protect work-life balance. Future research should evaluate the effectiveness of such corrective measures.

## Data Availability

The datasets are not available. Requests should be directed to jahernandezr@salud.madrid.org.
