# Supplementary material for: Burnout and workload among hematologists in the Community of Madrid (Spain): a survey based on the Copenhagen burnout inventory
Source: Front Public Health. 2026 Aug 24;14:1905977. doi: 10.3389/fpubh.2026.1905977 (PMC13547315; doi:10.3389/fpubh.2026.1905977)
Supplement: Supplementary file 1 [file Supplementary_file_1.docx]

**Supplementary Table S1. Professional and occupational characteristics of the participants.**

| **Characteristic** |  | **N=129**  **n (%)** |
| --- | --- | --- |
| Center funding type | Centers funded by public insurance | 107 (83) |
|  | Privately funded centers | 6 (5) |
|  | Mixed public-private centers | 16 (12) |
| Center complexity | High-complexity care centers | 83 (65) |
|  | Medium-complexity centers | 43 (33) |
|  | Low-complexity centers | 3 (2) |
| Professional position | Senior clinical management positions | 16 (12) |
|  | Staff specialists (attending physicians) | 101 (78) |
|  | Resident physicians | 12 (9) |
| Age group | <35 years | 42 (33) |
|  | 35-55 years | 59 (46) |
|  | >55 years | 28 (21) |
| Years of experience | <5 years | 26 (20) |
|  | 5–15 years | 49 (38) |
|  | 16-25 years | 29 (23) |
|  | >25 years | 25 (19) |
| Work setting | Clinical, alone | 55 (43) |
|  | Exclusively in the laboratory | 11 (9) |
|  | Exclusively in transfusion medicine | 7 (5) |
|  | Combined clinical and laboratory/transfusion | 56 (43) |

**Supplementary Table S2. Workload.**

| **Work** | | **N=129**  **n (%)** | **NA**  **n (%)** |
| --- | --- | --- | --- |
| Primary work activity (n = 101) | Patient care | 95 (94) | *28 (22)* |
|  | Teaching duties | 1 (1) |  |
|  | Research | 2 (2) |  |
|  | Management | 3 (3) |  |
| Weekly patient load (n = 98) | 1–25 patients/week | 24 (24) | *31 (24)* |
|  | 26–50 | 32 (33) |  |
|  | 51–75 | 24 (24) |  |
|  | 76–100 | 14 (14) |  |
|  | >100 | 4 (4) |  |
| Extra hours per week (n = 127) | None | 30 (24) | *2 (2)* |
|  | 1–5 h | 11 (9) |  |
|  | 6–10 h | 18 (14) |  |
|  | >10 h | 68 (54) |  |
| Main dedication of overtime hours (n = 98) | Patient care | 45 (46) | *31 (24)* |
|  | Teaching | 11 (11) |  |
|  | Research | 30 (31) |  |
|  | Management | 12 (12) |  |
| Telework (n = 102) | Yes | 45 (44) | *27 (21)* |
|  | No | 53 (52) |  |
|  | Other | 4 (4) |  |
| On‑call shifts per month (n = 129) |  |  |  |
|  | 1–5 shifts/month on call at the hospital | 35 (27) | *0 (0)* |
|  | >5 shifts/month on call at the hospital | 2 (2) |  |
|  | 1–5 shifts/month on call outside the hospital | 15 (12) |  |
|  | >5 shifts/month on call outside the hospital | 7 (5) |  |
|  | 1–5 shifts/month combining at the hospital and outside the hospital | 13 (10) |  |
|  | >5 shifts/month combining at the hospital and outside the hospital | 3 (2) |  |
|  | No on‑call shifts | 54 (42) |  |

**Percentages for response options are calculated over the number of respondents per item; the valid n is given for each variable. NA: not answered; percentage calculated over the total sample (N=129).**

**Supplementary Figure S1. Demographic characteristics of the participants.**

**Supplementary Figure S2. Workload.**

**Supplementary Figure S3. Time distribution by work area. Clinical setting.**

**Supplementary Figure S4. Perspectives of future variation of the time distribution by work area. Clinical setting.**

**Supplementary Figure S5. Time distribution by work area. Non-clinical setting.**

**Supplementary Figure S6. Perspectives of future variation of the time distribution by work area. Non-clinical setting.**

**Supplementary Figure S7. Summary totals by burnout domain. Personal and work-related burnout, n = 82; patient-related burnout, n = 79.**

**Supplementary Figure S8. Personal burnout. Frequency distribution of individual items. n = 82.**

**Supplementary Figure S9. Work-related burnout. Frequency distribution of individual items. n = 82.**

**Supplementary Figure S10. Patient-related burnout. Frequency distribution of individual items. n = 79.**
